# 结肠癌根治术后肠源性感染致脓毒症相关弥散性血管内凝血救治成功1例

**DOI:** 10.3760/cma.j.cn121090-20250104-00006

**Published:** 2024-12

**Authors:** 一娜 刘, 英健 梁, 健 张, 晓春 马

**Affiliations:** 中国医科大学附属第一医院重症医学科，沈阳 110001 Department of Critical Care Medicine, the First Affiliated Hospital of China Medical University, Shenyang 110001, China

患者，男，48岁，身高175 cm，体重68 kg，平素体健，2018年4月出现左上腹痛，间歇性发作，无恶心或呕吐，无腹胀或腹泻，伴发热，体温最高38 °C，自服抗生素（具体药物名称剂量不详）治疗，腹痛仍反复发作并逐渐加重。2018年6月于我院行肠镜检查发现结肠脾曲肿物，腹部MR平扫+增强见左上腹类圆形肿块，直径约10.5 cm、不均匀强化，与邻近胰腺脾脏分界不清。2018年8月9日行开腹手术，见肿瘤位于结肠脾曲近横结肠处，侵及脾脏，胰尾，胃大弯及侧腹壁，术中病理见结肠腺癌，低分化，侵破浆膜层伴大片坏死，侵及脾、胰腺组织，胃壁浆膜面见癌巢淋巴结，行左半结肠、脾、胰体尾及部分胃切除术。2018年8月11日体温38.3°C，应用厄他培南1.0 g每日1次静脉滴注抗感染，1 d后发热无缓解且WBC升高至22.22×10^9^/L，改为亚胺培南西司他丁1.0 g每日2次静脉滴注，2018年8月13日开始腹泻，24 h排3次黄色稀水样便总计600 ml，胃肠减压量增加至每日1400 ml，体温37.4 °C，心率上升至130次/min，血压120/79 mmHg（1 mmHg=0.133 kPa），全腹CT见肝周少量积气积液，结肠脾曲吻合口通畅，胰尾结构不完整、周围积气积液，小肠中段积液扩张伴多发气液平面影，联合万古霉素1.0 g每12 h 1次静脉滴注，补液。患者腹泻无好转，2018年8月14日体温升至40 °C，心率150次/min，血压85/45 mmHg，尿量减少至20 ml/h，呼吸困难，血氧饱和度90％，WBC 20.92×10^9^/L，中性粒细胞比例91.7％，C-反应蛋白292.3 mg/L，降钙素原（PCT）2.87 ng/ml。粪便球杆比1∶10。动脉血气pH 7.357，动脉血氧分压（PaO_2_）55 mmHg，动脉血二氧化碳分压（PaCO_2_）23.4mmHg，碱剩余−11.1 mmol/L，血乳酸3.8 mmol/L。因肠源性感染、感染性休克、Ⅰ型呼吸衰竭经口气管插管后转入ICU。

入ICU当晚进行有创呼吸机辅助通气，PC模式：吸气压力（PI）16 cmH_2_O，呼气末正压（PEEP）5 cmH_2_O，呼吸16次/min，吸入氧浓度60％。体温40 °C，心电监护：心率176次/min，血压85/60 mmHg，中心静脉压（CVP）6 mmHg，脉搏血氧饱和度90％，腹软，无肌紧张，肠鸣音5～6次/min，四肢末梢温度低、脉搏弱、稍苍白，脾窝引流少量淡血性液体，胰腺断端引流少量黄色液体，采血部位或动脉穿刺置管处血液凝固迅速。WBC 25.44×10^9^/L，粒细胞比例85.9％，HGB 107 g/L，PLT 329×10^9^/L；凝血酶原时间（PT）15.3 s，活化部分凝血活酶时间（APTT）49.4 s，纤维蛋白原5.18 g/L，D-二聚体>20 µg/ml，纤维蛋白降解产物（FDP）>150 µg/ml，抗凝血酶Ⅲ（ATⅢ）47％；动脉血气：pH 7.29，碱剩余−12.9 mmol/L，PO_2_ 89 mmHg（FiO_2_ 60％），PCO_2_ 25 mmHg，血乳酸3 mmol/L，血肌酐176 µmol/L，K^+^ 4.15 mmol/L，Na^+^ 137.2 mmol/L，Ca^2+^ 1.81 mmol/L，ALT 22 U/L，ALP 95 U/L，GGT 90 U/L，总胆红素13.5 µmol/L，直接胆红素13.2 µmol/L，ALB 20.1 g/L。急性生理与慢性健康（APACHE）Ⅱ评分22分，序贯器官衰竭（SOFA）评分9分，日本救急医学会（JAAM）评分4分，国际血栓形成与止血协会（ISTH）评分3分。予左氧氟沙星0.5 g每日1次联合万古霉素1.0 g每12 h 1次静滴抗感染，镇静镇痛、机械通气、液体复苏联合去甲肾上腺素抗休克，12 h后患者休克加重，心率151次/min，血压79/47 mmHg，需联合去甲肾上腺素1.5 µg·kg^−1^·min^−1^及垂体后叶素2 U/h维持血压，CVP 12 mmHg，尿量20 ml/h，12 h入液量3750 ml，出液量930 ml（尿量280 ml，胃肠减压量600 ml，腹腔引流总计50 ml），四肢末端苍白、冰冷、发绀, 体温38.4 °C，腹胀，血乳酸4.3 mmol/L，pH 7.15，碱剩余−7.7 mmol/L，氧合指数178 mmHg，血肌酐201 µmol/L，PT 15.2 s，凝血酶原活动度（PTA）76％，APTT 53.9 s，纤维蛋白原4.02 g/L，ATⅢ 39％，WBC 29.17×10^9^/L，中性粒细胞比例89.4％，HGB 93 g/L，PLT 195×10^9^/L，D-二聚体>20 µg/ml，FDP>150 µg/ml，JAAM评分5分，ISTH评分3分，C-反应蛋白256.7 mg/L，PCT 41.16 ng/ml，血培养厌氧瓶生长G^+^球菌，在液体复苏及血管活性药物基础上加用氢化可的松100 mg每日2次静滴抗休克，行CRRT，予普通肝素5 000 U/日+血必净100 ml每8 h 1次静脉泵入改善微循环。入ICU 24 h体温仍高达38.9 °C，腹胀无缓解，肠鸣音未闻及，24 h入液量6 410 ml，出液量2 260 ml（尿量330 ml，胃肠减压量1 100 ml，腹腔引流总计80 ml，CRRT脱水量750 ml），休克无缓解，心率140～150次/min，去甲肾上腺素1.5～2.0 µg·kg^−1^·min^−1^+垂体后叶素2～4 U/h维持血压于80～100/40～60 mmHg，CVP 12～14 mmHg，无尿，四肢紫绀加重、瘀斑范围扩大（图1），WBC 51.16×10^9^/L，C-反应蛋白259 mg/L，PCT 58.64 ng/ml，血乳酸4.2 mmol/L，PLT 83×10^9^/L，PT 20.2 s，PTA 44％，APTT>180 s，纤维蛋白原2.62 g/L，ATⅢ 28％，D-二聚体>20 µg/ml，FDP>150 µg/ml，JAAM评分8分，ISTH评分6分，因患者存在脓毒症相关弥散性血管内凝血（Disseminated intravascular coagulation，DIC）且合并肢体缺血，增加普通肝素至12 500 U/d持续静脉泵入抗凝，予前列地尔10 µg每日1次静滴改善微循环。入ICU 48 h体温38.2 °C，腹胀继续加重，膀胱压由14 cmH_2_O升至19 cmH_2_O，无排气或排便，胃肠减压血性液体，腹部切口见黄色脓液渗出伴有出血，血管导管留置处渗血，四肢紫绀加重（图1），24～48 h入液量3 720 ml，出液量2 360 ml（尿量210 ml，胃肠减压量1 200 ml，腹腔引流总计50 ml，CRRT脱水量900 ml），血培养回报耐甲氧西林表皮葡萄球菌，WBC 71.81×10^9^/L，C-反应蛋白271.8 mg/L，PCT 56.58 ng/ml，血清肌红蛋白19 900 ng/ml，PLT 13×10^9^/L，PT 20.6 s，APTT>180 s，纤维蛋白原2.21 g/L，D-二聚体>20 µg/ml，FDP 112.51 µg/ml，ATⅢ 27％，动脉血管超声：左侧尺、桡动脉远段彩色血流显示欠佳；右侧上肢肱动脉远段阻塞样病变，右侧尺、桡动脉彩色血流无显示，双侧下肢足背动脉内径较细，血流速度略减低。应用亚胺培南西司他丁1.0 g每8 h 1次+万古霉素1.0 g每12 h 1次+卡泊芬净70 mg每日1次静滴抗感染，普通肝素减量至8 750 U/d抗凝，输血浆400 ml、血小板1个治疗量，继续血必净及前列地尔静滴改善微循环、有创机械通气及CRRT支持治疗。入ICU 72 h，开始排黄色稀糊便，膀胱压力降至13 cmH_2_O，胃肠减压量减少至800 ml/d，48～72 h入液量3 370 ml，出液量2 600 ml（尿量200 ml，排便300 ml，胃肠减压量800 ml，腹腔引流总计100 ml，CRRT脱水量1 200 ml），切口流脓减少，体温37.1 °C～38.0 °C，WBC降至13.13×10^9^/L，PCT 13 ng/ml，休克纠正，肾功能尚未恢复，少尿状态，继续CRRT，但四肢末端发绀（图1），肌红蛋白5 670 ng/ml，切口及血管导管留置处仍有渗血，PLT 17×10^9^/L，PT 12.6 s，APTT 70.5 s，纤维蛋白原1.32 g/L，D-二聚体9.8 µg/ml，FDP 30.79 µg/ml，ATⅢ 35％，普通肝素减量至5 000 U/d抗凝，输血小板1个治疗量、冷沉淀10 U。入ICU 7 d后体温37 °C，无腹胀，每日排黄色糊状便500～1000 ml，经空肠管鼻饲百普力耐受良好，无休克表现，尿量增加至400 ml/d，无出血表现，四肢末端坏死范围局限、瘀斑范围缩小（图1），PLT 52×10^9^/L，PT 13.3 s，PTA 93％，APTT 68.3 s，纤维蛋白原2.74 g/L，D-二聚体5.64 µg/ml，FDP 16.26 µg/ml，ATⅢ 41％，血清肌红蛋白2 147 ng/ml，进入撤呼吸机流程，肾脏替代治疗间断进行。患者术后实验室检查结果及肝素剂量调整见表1。

**图1:**
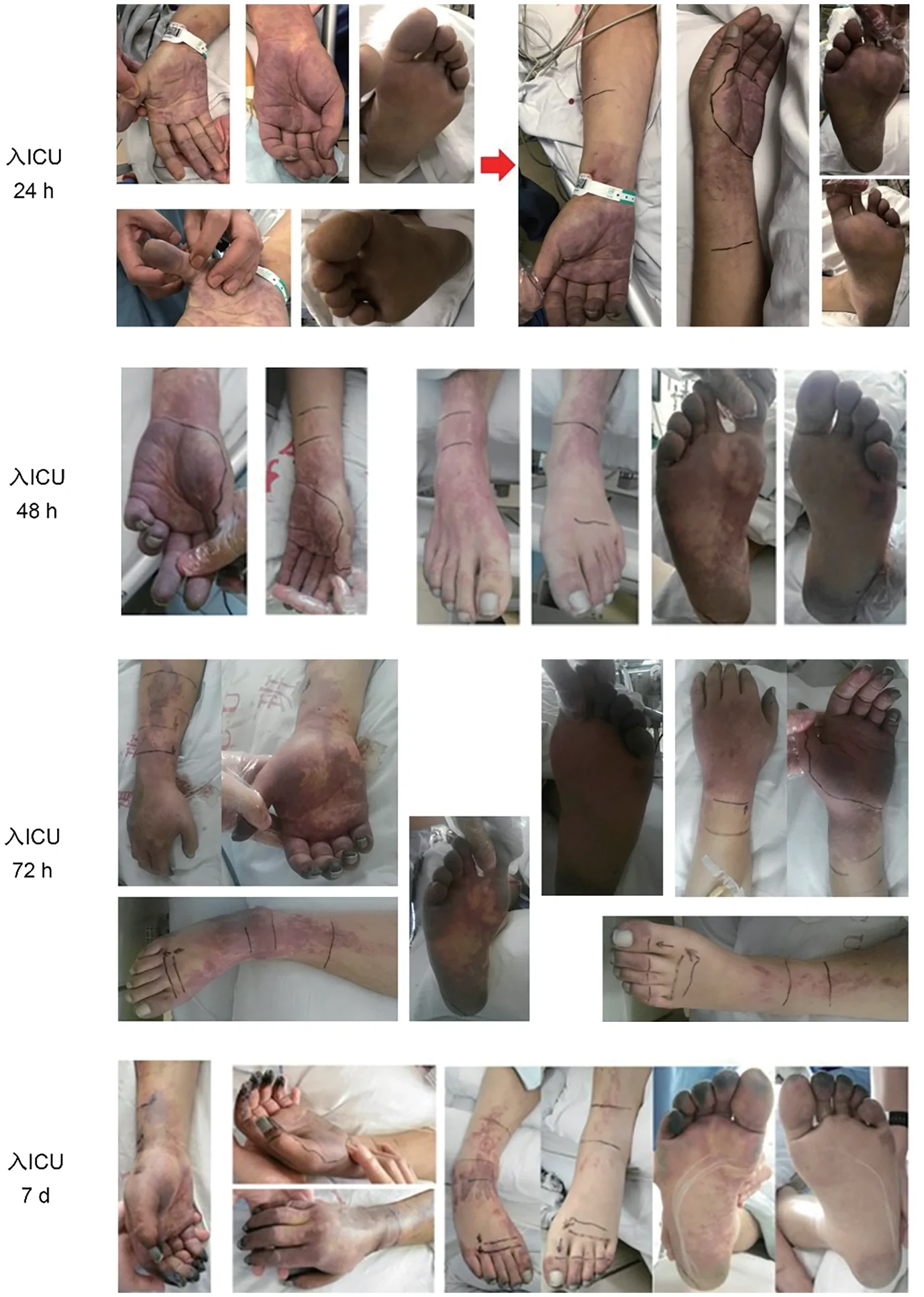
入ICU后不同时间点脓毒症相关弥散性血管内凝血合并肢体缺血表现

**表1:** 结肠癌根治术后肠源性感染致脓毒症相关弥散性血管内凝血患者出凝血功能实验室检查结果及肝素剂量调整

指标	入ICU后时间（h）					
	0	12	24	48	72	168
PLT（×10^9^/L）	329	195	83	13	17	52
APTT（s）	49.4	53.9	>180	>180	70.5	68.3
PT（s）	15.3	15.2	20.2	20.6	12.6	13.3
纤维蛋白原（g/L）	5.18	4.02	2.62	2.21	1.32	2.74
ATⅢ（％）	47	39	30	27	35	41
D-二聚体（µg/ml）	>20	>20	>20	>20	9.8	5.64
FDP（µg/ml）	>150	>150	>150	112.51	30.79	16.26
普通肝素抗凝量（U/d）		5000	12500	8750	5000	5000

**注** APTT：活化部分凝血活酶时间；PT：凝血酶原时间；ATⅢ：抗凝血酶Ⅲ；FDP：纤维蛋白降解产物

讨论：脓毒症DIC早期往往表现为高凝状态而非出血倾向，实验室检查可仅有血小板不同程度下降、D-二聚体及FDP升高，微血管血栓形成的征象包括组织器官灌注降低、肢端发绀及暴发性紫癜，肢体缺血常见于产毒素型细菌感染，可发生于感染性休克起病后48～96 h内，大剂量使用缩血管药物例如去甲肾上腺素可加重肢体缺血，导致对称性周围型坏疽，源于微血管血栓形成而非大血管阻塞，实验室检查见严重血小板减少、D-二聚体升高、PT延长及ATⅢ降低。脓毒DIC既有血栓形成又因血小板及凝血因子消耗导致出血是治疗难点，积极救治原发病的同时，应补充凝血底物纠正出血倾向并维持有效抗凝阻止过度的凝血反应激活，目前倾向脓毒症DIC合并显性血栓形成、暴发性紫癜、或肢端发绀应考虑肝素抗凝治疗，但使用肝素的具体时机及明确剂量没有达成共识。本病例因胃肠道手术后肠源性感染导致DIC，积极抗感染、机械通气及血液净化治疗为原发病缓解赢得了宝贵时间，感染及器官功能受累高峰时期肢体缺血进行性加重伴有出血倾向，早期使用普通肝素抗凝、血必净改善微循环可能是阻止微血管血栓过度形成的重要治疗手段，但发生出血时应用普通肝素必须密切观察出血表现有无加重，监测PLT、PT、纤维蛋白原、D-二聚体的动态变化，及时调整肝素用量并补充已消耗的凝血底物，可输注新鲜血浆、冷沉淀、纤维蛋白原及血小板。对于感染性休克患者特别是肺炎链球菌、葡萄球菌、大肠埃希菌、克雷伯菌、假单胞菌属引起的严重脓毒症，应警惕对称性周围型坏疽的发生，除尽快解决原发病之外，应积极进行液体复苏，避免使用过大剂量的缩血管药物并争取尽早停药，对于发生对称性周围型坏疽的病例，可使用肝素抗凝，尝试使用前列地尔、硝普钠、酚妥拉明扩血管治疗。

